# Increased Risk of Cardiovascular Diseases in Rheumatoid Arthritis: A Systematic Review

**DOI:** 10.7759/cureus.32308

**Published:** 2022-12-08

**Authors:** Hadi Farhat, Huma Irfan, Kanmani Muthiah, Namratha Pallipamu, Sogand Taheri, Suvedha S Thiagaraj, Twisha S Shukla, Sai Dheeraj Gutlapalli, Sheiniz Giva, Sai Sri Penumetcha

**Affiliations:** 1 Cardiology and Rheumatology, University of Balamand, Beirut, LBN; 2 Research, California Institute of Behavioral Neurosciences & Psychology, Fairfield, USA; 3 Research, Larkin Community Hospital, South Miami, USA; 4 Neurology, California Institute of Behavioral Neurosciences & Psychology, Fairfield, USA; 5 Internal Medicine, Franciscan Health, Lafayette, USA; 6 Internal Medicine, California Institute of Behavioral Neurosciences & Psychology, Fairfield, USA; 7 Medical Science, California Institute of Behavioral Neurosciences & Psychology, Fairfield, USA; 8 Pediatrics, California Institute of Behavioral Neurosciences & Psychology, Fairfield, USA; 9 Neonatology, Children's Health Ireland at Temple Street, Dublin, IRL; 10 General Medicine, California Institute of Behavioral Neurosciences & Psychology, Fairfield, USA; 11 General Medicine, Chalmeda Anand Rao Institute of Medical Sciences, Karimnagar, IND

**Keywords:** aortic stenosis, venous thromboembolism, cardiac death, ischemic heart disease, stroke, myocardial infarction, heart failure, atherosclerosis, cardiovascular disease, rheumatoid arthriitis

## Abstract

Rheumatoid arthritis (RA) is an autoimmune condition in which the body's joints are attacked by the immune system, leaving the patient disabled in severe cases, with irreversible joint damage and a lower quality of life. RA patients are more likely to develop cardiovascular (CV) disease, which increases their risk of morbidity and mortality. This study systematically reviews various CV diseases that might occur with RA including heart failure (HF), coronary artery disease, acute coronary syndrome, ischemic heart disease, stroke, cardiac death, venous thromboembolism, and valvular diseases. The relation between these complications and RA is specifically assessed. Systematic search was carried out on literature reporting the risk of each of the CV diseases in RA patients from databases in accordance with Preferred Reporting Items for Systematic Reviews and Meta-Analyses (PRISMA) guidelines. The databases searched were MEDLINE (through PubMed) and Google Scholar using a combination of keywords and medical subject headings (MeSH). Our keywords were mainly "cardiovascular diseases" and "arthritis and rheumatoid". We found a total of 33 articles reporting each CV comorbidity. Interestingly, a wide spectrum of CV diseases is reported in patients with RA. Many tools were implemented in the diagnosis of each disease such as carotid intima-media thickness for atherosclerosis and echocardiography for HF. We confirmed that RA is associated with an increased risk of different CV events, and prophylactic measures should be implemented.

## Introduction and background

Rheumatoid arthritis (RA) is an autoimmune disease in which a person’s immune system attacks the lining of joints throughout the body leading to disability and permanent joint damage and impairment of quality of life, especially in long-term and severe disease [1]. Other organs that RA may impact include the lungs, heart, blood vessels, skin, and eyes. Around one in two hundred persons worldwide suffer from RA, which affects women two to three times more when compared to men. Although it can affect anyone at any age, the peak onset is between the ages of 50 and 59 [2]. RA patients have an increased risk of cardiovascular (CV) disease occurring due to the inflammatory nature of the disease leading to higher morbidity and mortality rates [3-5]. CV events associated with RA range in a spectrum of severity. Some complications have low mortality such as hypertension (HTN) and dyslipidemia (DL) [6]. However, more severe complications include heart failure (HF), myocardial infarction (MI), coronary artery disease (CAD), and acute coronary syndrome (ACS) [7,8].

The diagnosis of HF is a clinical diagnosis and is categorized in many studies by assessing right ventricular and left ventricular systolic and diastolic functions [9,10]. A decrease in these parameters is highly suggestive of HF, especially in patients with RA. Traditional CV risk factors such as HTN, DL, and diabetes mellitus (DM) along with high concentrations of biomarkers associated with inflammation, fibrosis, congestion, and renin-angiotensin-aldosterone system activation all lead to a higher risk of HF [11]. Other CV events that rise with RA are based on the increased risk of atherosclerosis. Atherosclerosis, which can be diagnosed by an increase in intima-media thickness and an impaired nitric oxide bioavailability [1], elevates the likelihood of more severe complications like thrombosis [12], MI, and even stroke [13]. It is significant to note that coronary vasculitis [14], valvular lesions of the heart [15,16], and ischemic heart disease (IHD) [17] are other cardiac manifestations that can be found. Yiu et al. even found using computed tomography (CT) that RA is significantly associated with valvular calcifications such as the mitral valve and this independently predicted cases of premature atherosclerosis [18]. To comprehend the disease's impact on the cardiac structures of rheumatic patients, it may be helpful to summarize the data from recent investigations.

This systematic review intends to understand and affirm the hypothesis that there is a great diversity of CV events that might occur in RA and that different biological and molecular markers can assist in the diagnosis.

## Review

Methods

Search Strategy

Our systematic review was carried out in agreement with Preferred Reporting Items for Systematic Reviews and Meta-Analyses (PRISMA) [19]. The databases that were searched are the following: MEDLINE (through PubMed) and Google Scholar. The search method was tailored to each database and used a combination of keywords and medical subject headings (MeSH). The search term used in MEDLINE was ("Cardiovascular Diseases"[Majr]) AND "Arthritis, Rheumatoid"[Mesh]. Due to few data on PubMed regarding the relation between thromboembolic diseases and RA, keywords used to search on Google Scholar included "deep vein thrombosis", "pulmonary embolism", "venous thromboembolism", and "rheumatoid arthritis". In addition, manual searching was carried out by examining the references of pertinent review papers.

Study Selection

Studies that were incorporated into this systematic review were published from 1 January 2017 up to 30 July 2022. We included (i) cohort, cross-sectional, and case-control studies that concluded an association between CV diseases (HF, atherosclerosis, HTN, DL, MI, stroke, thrombosis, and valve disease) and RA, (ii) were published in English, and (iii) were performed on humans. Studies such as (i) case reports, commentaries, traditional reviews, and systematic reviews, those reporting on (ii) animal research, (iii) in a language other than English, and (iv) unavailable or retracted articles were excluded from this review. Two reviewers separately chose the papers that qualified for a full-text review based on their evaluations of the titles and abstracts of each study. Any differences of opinion were resolved through consensus. After applying the eligibility criteria to the chosen papers, they were further examined, and pertinent studies were then taken for a full-text review.

Data Extraction

To record data from the studies used for a comprehensive text evaluation, an Excel datasheet was employed. The following data were taken from each study: (i) study characteristics including the study design, the year of publication, the study population, and the country of the study; (ii) the type of CV event that occurred; and (iii) the diagnostic method of each disease.

Quality Assessment

The study quality was again assessed by two authors with the help of the Cochrane risk-of-bias tool and the Newcastle-Ottawa Quality Assessment Scale. The Cochrane tool is a widely utilized tool to appraise the quality of randomized trials. The tool is divided into five domains, and each has a risk-of-bias judgment at the end. These individual domain judgments are evaluated as “Low”, “High”, or “Some” concerns. We chose the articles that are judged to be at low risk of bias for all domains.

Regarding cohort, case-control, and observational studies, the Newcastle-Ottawa scale was utilized. The scale for case-control is divided into three categories: selection, comparability, and exposure. However, the scale for the cohort is divided into selection, comparability, and outcome. Several stars can be given to each article assessed. A total of 10 stars can be given for case-controls while a total of 13 stars can be given for cohort studies. We chose the case-controls that were given at least 7/10 stars (>70%) and the cohort studies that were given at least 9/13 stars (>69%).

Results

**Figure 1:**
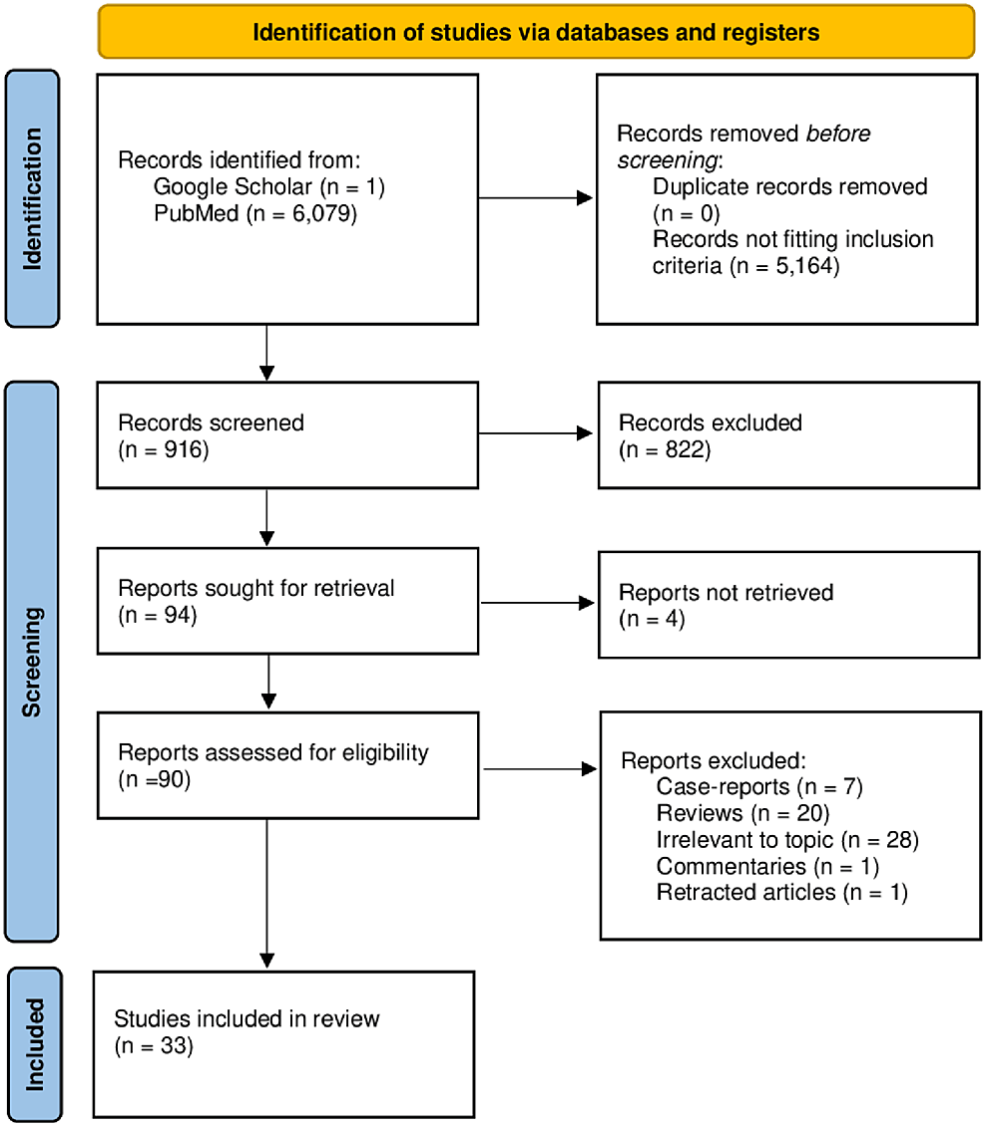
PRISMA diagram detailing the study identification and selection process. PRISMA: Preferred Reporting Items for Systematic Reviews and Meta-Analyses

There were no duplicates among the 916 records that our search produced (Pubmed-294 and manual hand-searching-one). Based on the title and abstract, another 822 were excluded. A full-text review of the remaining 94 papers was conducted. Thirty three of these papers were included in the final systematic review after meeting the inclusion criteria (Figure 1).

Study Characteristics

This systematic review only included English-language studies that involved human subjects and had been published during the previous five years. In the 33 articles included, there was a wide variety of CV diseases studied. Table 1 shows the study characteristics of each article included. These CV diseases might have common etiologies in RA patients and were distributed and assessed in the articles as follows: (i) Seven assessed the increased risk of arteriosclerosis and atherosclerosis; (ii) nine assessed the increased risk of HF; (iii) one assessed the increased risk of both atherosclerosis and HF; (iv) one assessed the increased risk of both HF and IHD; (v) one assessed the increased risk of both IHD and MI; (vi) two assessed the increased risk of ACS and atrial fibrillation; (vii) two assessed the increased risk of stroke; (viii) one assessed the increased risk of stroke, MI, and death; (ix) one assessed the increased risk of uncontrolled blood pressure; (x) one assessed the increased risk of cardiac arrest; (xi) two assessed the increased risk of venous thromboembolism (VTE); and (xii) one assessed the increased risk of aortic stenosis. 

**Table 1:** Study Characteristics. CV: Cardiovascular; IHD: ischemic heart disease; MI: myocardial infarction; HF: heart failure; IS: ischemic stroke; TIA: transient ischemic attack; CAD: coronary artery disease; VTE: venous thromboembolism; ACS: acute coronary syndrome

Study Number	Author	Year of Publication	Type of Study	Country	Number of Participants	Main CV Disease(s) Studied
1	Gomes et al., [20]	2017	Cross-sectional	Brazil	296	IHD and acute MI
2	Davis et al., [21]	2017	Prospective Longitudinal	United States of America	1551	HF
3	Holmqvist et al., [22]	2017	Prospective Cohort	Sweden	86,643	ACS
4	Meissner et al., [23]	2017	Case-control	Germany	12,354	Stroke
5	Aslan et al., [4]	2017	Observational Case-control	Turkey	280	Atherosclerosis
6	Bois et al., [16]	2017	Prospective Cohort	United States of America	68	Aortic stenosis
7	Khalid et al., [24]	2017	Prospective Cohort	Denmark	4,305,225	HF
8	Cioffi et al., [25]	2018	Prospective Cohort	Italy	392	HF
9	Løgstrup et al., [26]	2018	Prospective Cohort	Denmark	51,859	HF and IHD
10	Burggraaf et al., [27]	2018	Prospective Cohort	The Netherlands	212	Atherosclerosis
11	Adawi et al., [3]	2018	Prospective Cohort	Israel	40	Atherosclerosis
12	Biskup et al., [28]	2018	Case-control	Poland	103	Atherosclerosis and HF
13	Chen et al., [29]	2018	Prospective Cohort	Taiwan	3190	Recurrent Stroke in IS and TIA
14	Castro et al., [30]	2018	Observational Cross-sectional	Brazil	133	CAD
15	Targońska-Stępniak et al., [31]	2018	Prospective Cohort	Poland	64	Atherosclerosis
16	Dal Piaz et al., [32]	2019	Prospective Cohort	Italy	217	HF
17	Lee et al., [6]	2019	Cross-sectional	South Korea	25,828	CAD
18	Ruscitti et al., [33]	2019	Prospective Observational	Italy	841	Atherosclerosis
19	Bissell et al., [10]	2020	Cross-sectional	United Kingdom	102	HF
20	Ahlers et al., [7]	2020	Retrospective Cohort and Prospective Cohort	United States of America	19,778	HF
21	Dalbeni et al., [34]	2020	Prospective Longitudinal	Italy	137	Atherosclerosis
22	Li et al., [35]	2020	Prospective Cohort	United States of America	39,142	VTE
23	Björsenius et al., [5]	2020	Prospective Cohort	Sweden	86	Atherosclerosis
24	Çakmak et al., [36]	2020	Cross-sectional	Turkey	77	HF
25	Molander et al., [37]	2020	Prospective Cohort	Sweden	46,316	VTE
26	Elbadawi et al., [8]	2020	Observational	United States of America	123,783	Acute MI
27	Rodrigues et al., [38]	2020	Prospective Observational	Portugal	319	HF
28	Jang et al., [39]	2020	Prospective Cohort	South Korea	12,649	ACS, Atrial Fibrillation
29	Baviera et al., [13]	2021	Prospective Cohort	Italy	516,047	Stroke, MI, Death
30	Azpiri-Lopez et al., [9]	2021	Case-control	Mexico	112	HF
31	Ferreira et al., [40]	2021	Prospective Cohort	Portugal	355	HF
32	Almalki et al., [41]	2022	Cross-sectional	Saudi Arabia	834	Uncontrolled Blood Pressure
33	Hegazy et al., [42]	2022	Case-control	Denmark	35,195	Cardiac Arrest
Total					5,283,932	

The studies that were considered featured a geographically diverse population, with 20 coming from Europe, six from North America, five from Asia, and two from Latin America. Most of the studies included in our review were prospective cohorts (16 articles). However, five case-controls, six cross-sectional, and one retrospective cohort along with five prospective longitudinal and observational studies were also incorporated. Five studies had a sample size of <100, 14 with >100, and 14 others with >1000. Some of the articles with a sample size of >10000 were nationwide cohort studies. A total of 5,283,932 were assessed in the studies which is a significant population within the past five years.

The evaluation of the different CV diseases differed in each of the articles included. There were differences in the parameters used to assess each main CV complication and the diagnostic factors (imaging techniques) implemented along with the biomarkers measured. Atherosclerosis severity for instance was mostly estimated by the carotid intima-media thickness (cIMT) or the intima-media thickness measurements in general. One study also used the carotid segmental distensibility as an additional parameter while another used the local pulse wave pulse velocity. Other methods utilized were the ability to grow culture colony-forming units of endothelial progenitor cells which shows endothelial dysfunction. Regarding the imaging modalities, ultrasonography was the main tool that displayed the presence of plaques and the severity of atherosclerosis.

HF examinations also significantly differed among the included articles. The different parameters executed were ventricular end-systolic elastance, ventricular-vascular coupling ratio, right ventricular dysfunction, left ventricular systolic and diastolic dysfunction, and left ventricular mass index. Echocardiography was the main imaging technique, but cardiac magnetic resonance imaging was also implemented.

Regarding ACS and IHD, different diagnoses were carried out. Some articles assessed the risk of angina, MI, and atrial fibrillation in RA patients while others evaluated the possibility of ischemic stroke (IS) and even death. Another topic of interest was the possibility of an elevation in the risk of VTE such as deep vein thrombosis (DVT) and pulmonary embolism (PE), and rarely valve disorders such as aortic stenosis were highly associated with RA.

Discussion

Our review distinguishes itself from other studies in that it is the only one that is done in the past five years that examines a wide variety of increases in most CV events in RA patients. In general, most of the articles concluded an increase in the CV event studied whether it is atherosclerosis, HF, or even MI.

Atherosclerosis

As we stated before, atherosclerosis is a major comorbidity which was proven to be prevalent in RA patients by different means. A case-control study done by Biskup et al. was able to prove that the group of RA patients with ongoing mild disease activity had signs of accelerated atherosclerosis [28]. The length of the disease, patient age, and CV characteristics were found to be strongly correlated. Both RA patients and controls underwent a cIMT assessment. High-resolution B-mode ultrasonography was used to measure the cIMT. There were substantially higher cIMT values in RA patients compared to controls (0.83 (0.21) vs 0.62 (0.1) mm) and a higher incidence of atherosclerotic plaques (43 (61.4%) vs 10 (30.3%)). This was also proven by Björsenius et al. who did a prospective cohort of the 11-year period and found that when RA patients were compared to controls 11 years following diagnosis, they discovered that atherosclerosis development was more pronounced and that initial disease severity was correlated with the burden of atherosclerosis [5]. This was also assessed by the cIMT of the right carotid using ultrasound as the imaging technique.

In another prospective cohort done by Burggraaf et al. over three years, it was shown that RA patients with other comorbid conditions such as metabolic syndrome also have an increased risk of atherosclerosis [27]. In RA patients with metabolic syndrome compared to those without, baseline cIMT was substantially greater (0.619 (0.112) versus 0.557 (0.104) mm; p<0.001). The advancement of the cIMT was comparable after three years (0.043 (0.071) versus 0.043 (0.072) mm; p=0.96). Plaques were present in 12.9% to 23.5% of RA patients with metabolic syndrome throughout the three years. These results are also supported by Dalbeni et al. who performed a prospective longitudinal study and also showed that the development of subclinical atherosclerosis in RA patients is significantly influenced by both conventional CV risk factors and the inflammatory activity of rheumatic illness [34]. At baseline, carotid artery ultrasonography was used to screen for atheromatous plaques and quantify cIMT and carotid segmental distensibility in RA patients without a history of CV disease.

Moreover, Ruscitti et al., who performed a prospective observational study, revealed that participants who were prospectively monitored for three years had an elevated frequency and prevalence of both subclinical and symptomatic atherosclerosis [33]. Their results also found that subclinical atherosclerosis risk is higher in patients with type 2 DM, high blood pressure, and an increase in CRP levels. Another prospective cohort performed by Targońska-Stępniak et al. showed that considerable aggravation of atherosclerosis was discovered during a six-year course of established RA, shown by increased cIMT [31]. After six years, the mean cIMT value in RA patients was considerably greater [0.87 (0.21) vs 0.76 (0.15) mm; p<0.001], and it was shown that the proportion of patients with atherosclerotic plaques had increased.

It is significant to note that lipid parameters are increased in inflammatory diseases like RA. This predisposes to an increased risk of atherosclerosis in such diseases. For instance, lipoprotein (a) is an example of a lipid marker. Although there is a substantial hereditary component to lipoprotein (a), which has atherogenic and thrombogenic features, large concentrations of lipoprotein (a) have also been observed in the presence of inflammation, such as in RA [43].

All the mentioned studies confirm that atherosclerosis risk, confirmed by the measurement of cIMT, is increased in RA patients and prophylactic measures should be administered. Thus, blood tests of sugar levels and lipid panels showing cholesterol levels along with CT scans of major vessels should be performed early and more frequently to rule out atherosclerosis. Early pharmacological prophylaxis, such as statins, should be also considered in RA patients. 

Heart Failure

Patients with RA are at major risk of developing cardiac complications, especially HF. In a study done by Ahlers et al., they performed both a retrospective and another prospective cohort [7]. Their retrospective analysis showed that patients with RA had a 21% higher chance of having HF compared to those without the usual CV risk factors. The majority of the cases were HF with preserved ejection fraction. However, their prospective analysis focused on an inflammatory marker which is artemin in which a higher (worse) ventricular-vascular coupling ratio is related to higher levels of artemin. A case-control study done by Azpiri-Lopez et al. revealed that RA patients also had worse right ventricular function and right ventricular coupling with the pulmonary artery in comparison with the healthy controls [9]. This observation may explain the pathophysiology of HF in these patients. In addition to their results on atherosclerosis, Biskup et al. also showed that the ratio of early and late diastolic mitral inflow velocities, measured by standard and tissue doppler imaging in patients with high disease markers such as erythrocyte sedimentation rate, disease activity score-28 (DAS28) and high rheumatoid factor (RF)-immunoglobulin M (IgM), was decreased to less than one [28]. This shows that patients with higher disease markers have a higher risk of diastolic dysfunction than those with low markers.

Aside from echocardiography, cardiac magnetic resonance imaging was also used to diagnose HF. In the study done by Bissell et al., they showed that after accounting for conventional CV risk factors, patients with established RA who have no prior history of CV disease have evidence of impaired left ventricular systolic function and left ventricular mass index, the latter of which suggests cardiac pathology other than atherosclerosis in RA [10].

Rheumatologic markers can also be used to assess the severity of RA and consequently the risk of HF. This was confirmed by Çakmak et al. who measured RF, anti-citrullinated protein antibodies, and DAS28 with c-reactive protein and concluded that lower left ventricular systolic myocardial function and higher arrhythmia measures were related to the severity of RA [36]. Left ventricular myocardial dysfunction in RA patients was also studied by Cioffi et al. whose study was applied to normotensive RA patients [25]. Using echocardiography, they proved that in these patients, left ventricular systolic dysfunction is a potent predictor of a poor prognosis at the halfway point of the disease and that RA patients with HTN are at a greater risk of HF.

Furthermore, left ventricular diastolic function can also be affected in RA patients and can lead to HF. Dal Piaz et al. performed a prospective cohort also using Doppler echocardiography on patients with RA [32]. They also used the ratio between the peak velocity of the early diastolic “E” wave and the late diastolic “A” wave of transmitral flow to measure the degree of left ventricular diastolic function. They proved that at one-year follow-up, a large percentage of RA patients without obvious heart disease develop left ventricular diastolic dysfunction. Diastolic function was also assessed by Davis et al. who did a prospective longitudinal study [21]. Doppler echocardiography was also utilized to find the mean mitral inflow E/A ratio to show that diastolic dysfunction is a concern in RA patients. Rodrigues et al. also concluded that a 4% frequency of subclinical systolic function and a 13% prevalence of diastolic dysfunction were found in RA patients without established heart illness and that the most significant independent predictor of ventricular performance was aging [38].

Several protein biomarkers are associated with HF in patients with RA. In a prospective cohort underwent by Ferreira et al., they identified several biomarkers that increased in RA patients [40]. These biomarkers included adrenomedullin, placenta-growth-factor, tumor necrosis factor (TNF)-receptor-11A, and angiotensin-converting-enzyme-two. They also showed that traditional CV risk factors, such as DM, DL, and HTN, were more prevalent in patients with HF.

Several nationwide cohort studies also confirmed the increased risk of HF in these patients. Khalid et al. who performed a cohort study of the entire Danish population discovered that patients with RA had an elevated incidence of HF that was unrelated to any known risk factors [24]. This was also supported by Løgstrup et al. who also did a cohort of the Danish population and concluded that over 21 years, they report increased incidences of HF and IHD in patients with RA diagnosis [26].

These studies prove that systolic and diastolic heart failure is a major concern in RA patients. Therefore, more frequent echocardiography should be recommended for patients with RA, especially those with an increased risk for heart failure. 

Coronary Artery Disease/Ischemic Heart Disease and Acute Coronary Syndrome

CAD, IHD, and ACS were shown to be prevalent in RA patients. In a nationwide cross-sectional study done by Lee et al. in South Korea, patients with RA had a considerably higher frequency of CAD than the overall population [6]. Following the propensity score matching that they used, RA and CAD showed a strong correlation.

ACS and its relation with RA were evaluated by several studies. Elbadawi et al. underwent a 15-year observational nationwide analysis in the United States and deduced that the number of hospitalizations for acute MI-RA increased, primarily due to an increase in non-ST-elevation MI patients [8]. They also found that RA was independently linked to increased in-hospital mortality among patients with acute MI, especially in cases with ST-elevation MI. Moreover, Gomes et al. in a cross-sectional study concluded that acute MI was more common than prior investigations had found [20]. The variables associated with comorbidity included DM as one of the established risk factors and disease duration as one of the characteristics related to RA. Other nationwide cohorts also evaluated the ACS-RA correlation. Holmqvist et al., in a nationwide study on the Swedish population, showed that about 40% more people with RA had an overall risk of developing ACS than the general population [22]. They also stated that patients with DAS28 ≥ 3.2 at RA diagnosis and patients who were RF-positive were the lone category of patients associated with increased risk. Another nationwide cohort done by Jang et al. in South Korea also proved that compared to the matched controls, elderly individuals with RA had a greater risk to develop ACS [39].

Patients with RA have been also shown to be at increased risk of cerebrovascular diseases. Chen et al. conducted a cohort study and demonstrated that RA markedly enhanced the probability of recurrence following an IS and transient ischemic attack (TIA) [29]. Patients with RA had a 37% higher risk of recurrent stroke and a 41% higher risk of recurrent IS/TIA. They also found that with an elevated triglycerides/ high-density lipoprotein cholesterol ratio came an increased risk of recurrent IS/TIA. Meissner et al. in a large cohort concluded that age and smoking, two well-recognized risk variables, as well as hyperlipoproteinemia and diminished physical function, were linked to a higher risk of stroke in RA patients [23]. Moreover, they found that patients who had another serious adverse event within 30 days of their stroke had the greatest incidence rate of stroke.

Some studies have associated RA with an increased risk of cardiac arrest and death. The cohort study done by Baviera et al. depicted that in comparison to the general population, RA was linked to a considerably increased risk of death, stroke, and MI [13]. Along with all cerebrovascular and CV events, the RA cohort also had a markedly increased risk of HF and peripheral vascular disease. This was also emphasized by Hegazy et al. in a nationwide case-control study [42]. They claimed that RA was linked to a higher likelihood of cardiac arrest occurring outside of a hospital. This elevated rate continued even after taking into account the usual risk factors for out-of-hospital cardiac arrest, which were present in women but not in men. Patients with and without CV comorbidities did not have significantly different out-of-hospital cardiac arrest rates.

Thus, tests that monitor CAD should be more frequently implemented in RA patients to rule it out. These tests include the exercise stress test, cardiac CT, and cardiac angiogram. 

Other Cardiovascular Complications

Patients with RA have been also found to be at an increased risk for other CV events. These events include uncontrolled HTN, systemic arterial HTN, pulmonary arterial HTN, VTE, and valve diseases like aortic stenosis. Almalki et al. performed a cross-sectional study on hypertensive adults with RA [41]. In Saudi Arabia, their study looked at the prevalence of uncontrolled blood pressure and identified the risk factors associated with it. They calculated the prevalence of uncontrolled blood pressure in the research sample to be 31.65%. Systemic arterial HTN and DL were also reported at a high frequency in a cross-sectional study by Castro et al. [30]. On top of that, pulmonary arterial HTN was also shown to be prevalent in rheumatic diseases including RA. Chen et al. found that that Interstitial lung disease, congestive heart failure, valvular heart disease, and thyroid disorders were all strongly related to pulmonary arterial hypertension [44]. 

VTE can be manifested as DVT and if left untreated can lead to PE. Li et al., in a prospective cohort, proved that when compared to the general population without RA, RA was linked to a higher incidence of VTE, PE, and DVT occurrences [35]. These results are also confirmed in a nationwide cohort study done in Sweden by Molander et al. [37]. They discovered a significant correlation between the risk of VTE and the DAS28-measured RA disease activity and that high DAS28 increased risk for PE twice as much as it did for DVT. 

These data suggest that there should be more frequent usage of doppler ultrasound and D-dimer tests in RA patients to assess them for DVT and PE in order to provide therapy accordingly. 

Finally, cases of valvular diseases have been shown to arise in RA patients. We found a study done within the past five years that evaluated aortic stenosis in these patients. Bois et al. used echocardiography to investigate aortic stenosis in RA patients [16]. They concluded that the proportion of RA patients who also have aortic stenosis will probably keep rising. This shows that echocardiograms and valvular analysis should be assessed in patients with RA to rule out valve abnormalities. 

Limitations

Even though our study offered many strengths, there were some limitations. First, we were able to find and include only two articles that assessed the increased risk of VTE, DVT, and PE in RA patients. We also found only one article that covered valvular disease risks such as aortic stenosis. More studies should be encouraged in this matter, and other valvular diseases such as mitral stenosis and mitral and aortic regurgitation should be considered.

Moreover, many of the articles included patients in their population who already had CV risk factors before the initiation of the study. This decreases the credibility of the study since it may neglect RA as the main cause of increased CV diseases in these patients.

## Conclusions

This systematic review looked into the increased risk of various CV events in RA. Since patients with RA are susceptible to early atherosclerosis, DL, HTN, and various risk factors, they are more prone to HF, IHD, CAD, ACS, VTE, and even valvular disorders. These risks have been proven by a lot of studies and should be taken seriously. Considering early prophylaxis in these patients is advisable, and hospitals and healthcare centers should incorporate protocols to consider these complications while handling patient care and treatment. We propose that early and more frequent screening for these CV complications should be implemented. Screening for more severe complications such as ACS, stroke, and HF should be prioritized over less severe ones. Cardiac echocardiograms, lipid profiles, CT angiograms, and doppler ultrasound have to be performed in the early stages of the disease, and follow-up with these patients should be ordered to prevent the progression of these complications if diagnosed. 
